# Intranasal administration of erythropoietin rescues the photoreceptors in degenerative retina: a noninvasive method to deliver drugs to the eye

**DOI:** 10.1080/10717544.2018.1556361

**Published:** 2019-02-11

**Authors:** Ye Tao, Chong Li, Anhui Yao, Yingxin Qu, Limin Qin, Zuojun Xiong, Jianbin Zhang, Weiwen Wang

**Affiliations:** a Department of Ophthalmology Key Lab of Ophthalmology and visual science, Chinese PLA General Hospital, Beijing, PR China;; b Department of Physiology, Basic Medical College, Zhengzhou University, Zhengzhou, PR China;; c Department of Neurosurgery, Chinese PLA General Hospital, Beijing, PR China;; d Department of Neurosurgery, Central Hospital of Wuhan Tongji Medical College Huazhong University of science and technology, Wu Hang, PR China;; e Department of Occupational and Environmental Health Ministry of Education Key Lab of Hazard Assessment and Control in Special Operational Environment School of Public Health, Fourth Military Medical University, Xi’an, China;; f Department of Neurosurgery and Institute for Functional Brain Disorders, Tangdu Hospital Fourth Military Medical University, Xi’an, PR China

**Keywords:** Intranasal delivery, neural degeneration, therapeutics

## Abstract

Inherited retinopathies typically lead to photoreceptor loss and severe visual impairments in the subjects. Intranasal administration is an efficient approach to deliver therapeutic agents to the targeted tissue. The present study is designed to deliver the erythropoietin (EPO) into the N-methyl-N-nitrosourea (MNU) induced mice, a pharmacological retinopathy model via intranasal or intravenous route. The mice were then subjected to bioavailability assay and therapeutic effects evaluation. Our results showed that the intranasal delivery of EPO is effective to alleviate the morphological disruptions in the MNU induced mice. The intranasal delivery of EPO also ameliorated the visual impairments in the MNU induced mice. Immunostaining experiment showed that both the M-cone and S-cone populations in the degenerative retinas are rescued by the intranasal delivery of EPO. In particular, the M-cone photoreceptors in dorsal-temporal (DT) quadrant and the S-cone photoreceptors in ventral-nasal (VN) quadrant were preferentially preserved by the intranasal delivery of EPO. Mechanism studies showed that the intranasal delivery of EPO could the modulate apoptosis and restrict oxidation in the degenerative retina. Compared with intravenous delivery, the intranasal delivery led to the significantly higher EPO concentration in the retina. The intranasal delivery resulted in more potent protection and had less erythropoiesis-stimulating activity than the intravenous delivery. Our results suggest that the intranasal administration is a noninvasive and efficient approach to deliver EPO into the retinas. These findings lay the groundwork for further intranasal administration of EPO in ophthalmological practice.

## Introduction

Retinitis Pigmentosa (RP) comprises a family of hereditary retinal dystrophies that are characterized by the progressive photoreceptor degeneration and severe visual impairments. The worldwide prevalence is ∼1 in 4000 individuals with a total of 1.5 million affected individuals (Hartong et al., 2006). One puzzling aspect of RP concerns the enormous heterogeneity, as more than 180 genetic mutations affecting the phototransduction cascade could result in RP. Currently, experimental therapies for RP include pharmacologic intervention, gene rectification, stem cell transplantation, and visual prostheses (Souzeau et al., 2018; Moreno et al., 2018). These breakthroughs highlight the need for advanced drug-delivery methods which are capable of increasing the stability and bioavailability of therapeutic agents. Admittedly, this work is challenging because several physiological barriers restrict the transport of executors to the posterior segment of eye. In clinical practice, the intravitreal injection renders increased drug concentrations in the retina and circumvents these unwanted systemic effects. However, the chronic circle of retinopathy necessitates frequent pharmacological interventions. The repeated intravitreal injection would give rise to severe surgical complications such as retinal detachment, endophthalmitis, and elevated intraocular pressure(Fangueiro et al., 2016; Thrimawithana et al., 2011). Therefore, noninvasive delivery methods are necessary to afford safe and efficient protection.

The intranasal (INas) administration is an emerging approach to deliver therapeutic agents to the central nervous system (CNS). Several insightful studies have shown the intranasal administration of drug results in protection on the CNS. However, these protective effects are not seen after intravenous (IVen) administration of the same drug (Pietrowsky et al., 1996). It is highly possible that these drugs are transported via a direct nose-to-brain pathway which bypasses the systemic circulation. Later pharmacological investigations have not only studied the therapeutic effects of the INas delivered drug, but also analyzed their specific concentrations in the CNS tissue. In this context, this versatile route is developed into a promising approach in the treatment of CNS disorders, such as cerebral ischemia, Alzheimer’s disease, traumatic brain injury, Parkinson’s disease and brain tumors (Capsoni et al., 2009). Some candidate therapeutics such as the NGF, interferon, insulin-like growth factor-I, curcumin, and erythropoietin (EPO) have been successfully delivered to the brain of animal models and human patients via INas delivery (Garcia-Rodriguez & Sosa-Teste, 2009; Illum, 2002). Of note, the EPO has demonstrated exciting therapeutic potency against chronic neurodegenerative disorders. EPO is initially recognized as a glycoprotein to stimulate the generation of erythrocytes. Accumulating evidence shows that EPO also governs the development, differentiation, and survival of neural cells by modulating apoptotic pathways (Osikov et al., 2015). Pioneering studies have translated the EPO induced favorable effects into clinical utility. According to the information of National Institutes of Health, more than 60 clinical trials have been launched to evaluate the EPO induced protective effects on neural diseases (Maiese, 2016). However, the systemic EPO administration always elevates the hematocrit value and further increases the risk of thrombosis (Wiessner et al., 2001). The erythrogenesis and proangiogenic effects of EPO impose tremendous limitations on clinical application, especially for these patients with diabetes or hypertension. Therefore, a novel delivery approach is needed to enhance therapeutic efficacy and limit the undesired side effects. Several lines of evidence show that the INas delivered EPO has minimum systemic effects (Genc et al., 2011). In both rodents and nonhuman primates, the INas delivered EPO could reach the CNS faster than those via IVen route (Garcia-Rodriguez & Sosa-Teste, 2009). These findings highlight the possibility that the INas pathway might be advantageous over conventional delivery approaches in EPO therapy.

As a critical component of the visual circuit, the retina is responsible for the conversion of light stimulus into electrical spikes that are subsequently interpreted in the cortex as vision (Balasubramanian & Sterling, 2009). Hitherto, the effects of INas delivered EPO on the retina remain to be explored. The present study is designed to deliver the EPO into the N-methyl-N-nitrosourea (MNU) administered mice via INas route (Tao et al., 2015; Kinoshita et al., 2015). The N-methyl-N-nitrosourea (MNU) administered mice serves as a pharmacologically induced RP model with rapid progress rate. It becomes evident nowadays that the MNU-induced photoreceptor degeneration is caused by apoptosis. After systemic administration, MNU induces DNA alkylation and paralyzes instinctive the base excision repair machinery within photoreceptors nucleus (Meira et al., 2009). Hence the suicidal cascade is activated and the affected photoreceptors would turn into apoptosis in parallel with terrible visual impairments. The MNU induced RP model is characterized by the extinguished electroretinogram (ERG) waveform, disorganized retinal structure and secondary neuronal remolding (Tao et al., 2015). Many of these hallmarks compare well with those found in human RP patients. Our results suggest that the INas delivery of EPO is effective to alleviate the retinopathy of the MNU administered mice. The INas route had significantly higher efficiency to deliver EPO to mouse retina compared with the IVen route. Moreover, the INas deliver approach had less erythropoiesis-stimulating activity. These findings lay the groundwork for future INas delivery of EPO in ophthalmological practice.

## Method and materials

### Animal administration and study design

The C57BL/6 mice (8 weeks old with both sexes, Animal Center of PLA General Hospital, Beijing, China) were housed in the air-conditioned facility (room temperature: 18 °–23 °C, humidity: 40–60%). The illumination in the animal facility was 85 lux (12 h dark/light cycle with lights on at 07:00 and off at 19:00); All animals were handled in accordance with the Association for Research in Vision and Ophthalmology’s Statement for the animal usage. Whole study protocol was reviewed and approved by the institutional animal care and use committee of the General Hospital of PLA. The experimental animals were divided randomly to four groups: (1) Normal control group: C57 mouse without any pharmacological administration; (2) MNU group: C57 mouse received an intraperitoneal injection of MNU; (3) Intranasal (INas) treated group: C57 mouse received INas delivery of EPO after MNU administration. (4) Intravenously (IVen) treated group: C57 mouse received IVen delivery of EPO after MNU administration. The MNU solution (5 mg/ml) was prepared by dissolving reagent in physiological saline containing 0.05% acetic acid. In order to establish the retinal degeneration model, the mice received a single intraperitoneal injection of MNU (60 mg/kg; Sigma-Aldrich Corp., St. Louis, MO, USA). For INas administration, mice were anesthetized with 2% sodium pentobarbital (2.5 ml/kg, Sigma-Aldrich Corp, IL, USA) and were placed in a supine position (Capsoni et al., 2009). The Recombinant human EPO (Amgen, CA, USA) was diluted in phosphate-buffered saline (PBS; pH 7.0). EPO solution (12 µl, 100UI) was administered by pipette in nose drops (2 µl/drop) over a 12 minutes period, alternating between each nare. During the administration procedures, the mouth and the opposite nares of mouse were closed so that the drops could be naturally inhaled high into the nasal cavity. The dose and frequency of EPO administration followed a previous pharmacological study (Fletcher et al., 2009). For IVen administration, mice were placed in a supine position and the EPO solution (200 µl, 100UI) was injected into the vena caudali. In both INas and IVen administration, the first dose of EPO was given two hours after MNU administration, while the second and third doses were given respectively 24 h and 48 h after the MNU administration.

### Optokinetic behavioral test

**Figure 1.**
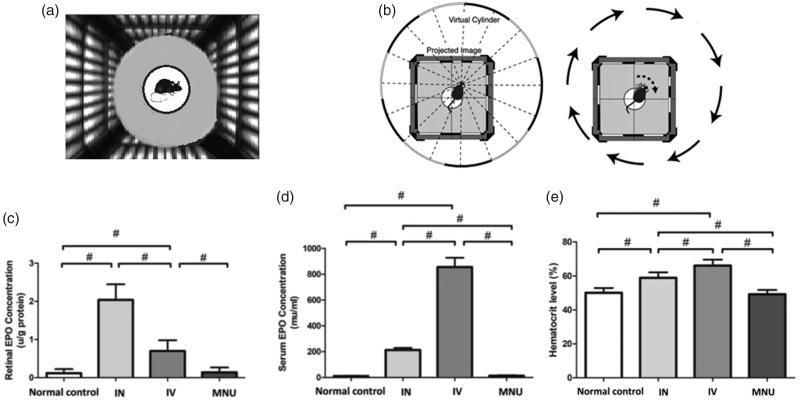
(A) During the optokinetic behavioral test, the mouse was placed on a platform of the text box. Virtual cylinders were projected on the wall of the box and they turned either in clockwise or counter-clockwise direction for the mouse to track. (B) The optokinetic behavioral test was performed using a two-alternative forced choice paradigm. The right picture shows an example of track response when the virtual cylinders are turning in clockwise direction. (C) The EPO expression levels in the retinas of different animal groups. (D) The EPO expression levels in the serum of different animal groups. (ANOVA analysis followed by Bonferroni's post-hoc analysis was performed, #*p* < .01, for differences between groups; *n* = 10).

One week after MNU administration, the optokinetic behavioral test was performed using a two-alternative forced choice paradigm as described previously(Kretschmer et al., 2015). Ten mice from each animal group were placed on a platform of the test box (Figure 1(A)). Virtual cylinders were projected on the wall of the box. These cylinders turned either in clockwise or counter-clockwise direction for the mouse to track. Figure 1(B) shows a typical track response of the mouse when the virtual cylinders are turning in clockwise direction. The stepwise correction was used to determine the response thresholds. The initial stimulus of visual acuity measurements was set as 0.200 cyc/deg sinusoidal pattern with a fixed 100% contrast. The initial pattern in contrast sensitivity measurements was set as 100% contrast, with a fixed spatial frequency of 0.128 cyc/deg. All patterns were presented at a speed of 12 degrees/s with the mean luminance of 70 cd/m^2^.

### ERG examination

One week after MNU administration, the ERG examination was carried out to evaluate the visual function of experimental animals. Ten mice from each animal group were dark adapted for 12 hours before examination. They were anesthetized by an intraperitoneal injection of ketamine (80 mg/kg) and chlorpromazine (15 mg/kg, Shengda, Jilin, China). Their pupils were dilated with 1% atropine and 2.5% phenylephrine hydrochloride (Xing Qi, Shenyang, China). The RETIport system (Roland Consult, Germany) with custom-made chloride silver electrodes were used for the recording. Flash responses were recorded through corneal electrodes, with the reference electrode placed in the subcutaneous space of the cheek, and the ground electrode clipped to the earlobe respectively. Scotopic ERGs were recorded at a stimulus intensity of 0.5 log cd-s/m^2^ with the inter-stimulus intervals of 30 s. After scotopic ERGs measurement, the mice were light adapted for 10 min at the intensity of 30 Ganzfeld cd-s/m^2^. White flash (1.48 log cd-s/m^2^) was delivered from a Ganzfeld integrating sphere to stimulate the response. For waveform analysis, the amplitude of a-wave was defined as the distance from baseline to a-wave trough, while the amplitude of b-wave was measured as the distance between trough and peak of each waveform. The recorded signals were amplified and filtered (1–300 Hz) by band-pass. Totally 60 photopic responses and 10 scotopic responses were collected and averaged for b-wave analysis.

## Spectral-Domain optical coherence tomography (SD-OCT)

After ERG examination, the SD-OCT assay was performed to analyze the retinal architecture while the mice were still anesthetized. These mice were subjected to a repeated dilation to avoid pupil retraction. Subsequently, they were transferred to the recording plane of an ultrahigh-resolution instrument (Bioptigen, Durham, NC, USA). A corresponding box was centered on the optic nerve head. Eight measurements at the same distance (3 mm) from each other were conducted. Three hundred linear B-scans were obtained, and 30 averaged images were captured to achieve a better resolution. The cross-sectional images were analyzed with the InVivoVueTM DIVER 2.4 software (Bioptigen, Durham, NC, USA).

### Quantification of EPO concentration and hematocrit level

Ten mice from each animal group were sacrificed and their eyecups were enucleated one week after MNU administration. The retina was carefully isolated from anterior segments under dissecting microscope. The retina tissue was homogenized in radio-immunoprecipitation assay (RIPA) lysis and extraction buffer, and then was sonicated at 0.5 Hz for 50 s (50-watt sonicator, Sonics & Materials, Danbury, CT). The serum EPO concentration was analyzed by an ELISA kit (R&D Systems, Genetimes Technology, Inc., Shanghai, China) according to the manufacturer’s protocol. The retinal EPO level was normalized by the total protein content, as determined by a bicinchoninic acid kit (PRIERCE Bio, Rockford, IL, USA). Data are present as U/g protein or mU/ml. Hematocrit level was quantified using a microcapillary centrifugation (Sysmex analyzer, Japan).

### Histology assessment and immunohistochemistry

Retinal sections and whole-mount preparations were prepared following a previously described method (Curcio et al., 1987). Briefly, ten mice from each animal group were sacrificed.

The enucleated eyecups were immersed in a fixative solution containing 4% paraformaldehyde (Dulbecco's PBS; Mediatech, Inc., Herndon, VA) overnight at 4 °C. The anterior segments were cut off and the retinas were rinsed with PB (phosphate buffer), dehydrated in a graded ethanol series, and embedded in paraffin wax. Retinal sections (thickness of 5 μm) were cut vertically and then were stained with hematoxylin and eosin (HE). The thicknesses of the ONL were measured at 250 µm intervals along the vertically superior-inferior axis by a single observer in a masked fashion. Data from three sections (selected randomly from six sections) were averaged for each eye. The ONL thicknesses of ten eyes were averaged for each animal group. On the other hand, retinal whole-mount preparations were generated by first removing the optic nerve head and then carefully separating the neuroretina from the eyecup. Subsequently, the retinal specimens were rinsed in 0.01 M PBS, permeabilized in 0.3% Triton X-100, and blocked in 3% BSA for 1 hour at room temperature. The peanut agglutinin (PNA) conjugated to an Alexa Fluor 488 (1: 200, L21409, Invitrogen, USA), S-cone opsin, or M-cone opsin antibodies (1: 400, Millipore, MA, USA) were diluted in 0.1% Triton X-100 and 1% BSA in PBS, and then were incubated with retinal specimen overnight at 4 °C. After 3 rinses with PBS, the retinal specimens were incubated in Cy3-conjugated anti-rabbit IgG (1:400, Jackson ImmunoResearch Laboratories, USA) and DAPI. Retinal specimens were rapidly rinsed with 0.01 M PBS five times and then were coverslipped with anti-fade Vectashield mounting medium (Vector Labs, Burlingame, CA, USA) for photographing. Fluorescence was analyzed with the Zeiss LSM 510 META microscope (Zeiss, Thornwood, NY, USA) fitted with Axiovision Rel. version 4.6 software (Carl Zeiss AG Manufacturing company, Oberkochen, Germany). All fluorescent images were captured using identical exposure settings. The cone numbers of four 420 × 420 μm squares which located 1 mm dorsal, temporal, ventral, and nasal to the center of the optic nerve were determined.

### TUNEL assay

Typically, the MNU induced activation of apoptotic cascade would peak at P3, and then return to the baseline around P7 (Chen et al., 2014). Accordingly, we performed the TUNEL assay at P3 to capture the most evident changes in apoptotic signaling. Terminal deoxyuridine triphosphate nick-end labeling (TUNEL) assay was performed on paraffin sections as described previously (Yoshizawa et al., 2000). The in situ cell death detection POD Kit (Roche Diagnostics GmbH, Mannheim Germany) was used according to the manufacturer’s protocol. Subsequently, the TUNEL sections were counterstained with DAPI, mounted on slides, and then visualized with confocal microscopy (LSM510, Zeiss, Oberkochen, Germany). The apoptotic index (AI) of the ONL was calculated on the basis of cell numbers (number of TUNEL-positive nuclei/total number of photoreceptor cell nuclei × 100).

### Assay of superoxide dismutase (SOD) activity and malondialdehyde (MDA) content

Three days post-MNU administration ten mice from each animal group were sacrificed and their eyecups were enucleated. The retinal SOD activity and MDA content of experimental animals were examined as described previously (Tao et al., 2018). The SOD activity was examined with the Assay Kit-WST (Jiancheng Biotech Ltd., Nanjing, China). MDA content was assessed using a total bile acid colorimetric assay under the guidance of the manufacturer’s protocol (Jiancheng Biotech Ltd., Nanjing, China).

### Quantitative reverse transcription-polymerase chain reaction (qRT-PCR)

Three days post-MNU administration, the mRNA transcripts levels of Bax, Bcl-2, Caspase-3, C/EBP-homologous protein (CHOP) in retinas were measured by qRT-PCR. Total RNA was isolated from mouse retinal tissues (Trizol Reagent; Invitrogen), and cDNA was synthesized usingμMACS™ DNA Synthesis kit (Miltenyi Biotech GmbH, Bergisch-Gladbach, Germany). All quantitative PCR reactions were performed via a real-time CFX96 Touch PCR detection system (Bio-RadLaboratories, Reinach, Switzerland). The primers used in qRT-PCR were: Bax: 5′-AGCTCTGAACAGATCATGAAGACA-3′ (forward) and 5′-CTCCATGTTGTTGTCCAGTTCATC3′ (reverse); Bcl-2:5′-GGACAACATCGCTCTGTGGATGA-3′ (forward) and 5′-CAGAGACAGCCAGGAGAAATCAA-3′ (reverse); Caspase-3:5′-TGTCGATGCAGCTAACC-3′ (forward) and 5′-GGCCTCCACTGGTATCTTCTG-3′ (reverse); CHOP: 5′-CTGCCTTTCACCTTGGAGAC-3′ (forward) and 5′-CGTTTC CTGGG GATGAG ATA -3′ (reverse). The relative expression levels were normalized and quantified to obtain the ΔΔCT values (DATA assist Software v2.2, Applied Biosystems).

### Isolation of mitochondria and analysis of manganese-superoxide dismutase (Mn-SOD)

Retinal tissue was washed and homogenized using buffer A [0.3 phenylmethylsulfonyl fluoride, 1 orthovanadate, 1 NaF, 1 EDTA, 10 Tris-HCl, 250 sucrose (in mmol/l)], followed by serial centrifugations (700 × g, and 10000 × g for 30 min respectively). The resulting supernatant was collected and defined as the cytosolic fraction, while the mitochondrial pellet was washed, collected and lysed in mitochondria-specific buffer B [0.3 PMSF, 1 NaF,1 orthovanadate, 10 EDTA, 20 Tris-HCl, 150 NaCl (in mmol/l), 1% NP-40]. Then the supernatant was collected and subjected to Mitochondrial Mn-SOD analysis. The Mn-SOD activity was measured using commercially available kits under the guidance of the manufacturer’s instructions (Beyotime Biotechnology, Shanghai, China). The Mn-SOD activity was expressed as relative percent of control.

### Statistical analysis

Statistical difference between experimental animal groups was processed using the ANOVA analysis followed by Bonferroni's post-hoc analysis. All the *p* values are presented as mean ± standard deviation (SD). *p* value <.05 was considered significant.

## Results

### Assessment of EPO levels in the experimental animals

No clinical sign or systemic symptom was evident in the EPO treated animals during the whole experiment process. The EPO levels of mice were assessed using an ELISA kit. The retinal EPO concentration in the MNU group was not significantly different from that in the normal control (*p* > .05; *n* = 10), suggesting the MNU administration alone would not affect retinal EPO level in normal mice (Figure 1(C)). The retinal EPO concentration in the IVen administrated group was significantly higher than that in the normal controls (*p* < .01, *n* = 10). The retinal EPO concentration in the IVen administrated group was significantly lower than that in the INas administrated group (*p* < .01, *n* = 10). Moreover, the serum EPO levels in the INas and IVen administrated groups were both significantly higher compared with the MNU group (*p* < .01, *n* = 10; Figure 1(D)). Particularly, the serum EPO concentration in the INas administrated group was significantly lower than that in the IVen administrated group (*p* < .01, *n* = 10). Compared with the IVen delivery, the INas administration rendered higher EPO level in the retina and lower EPO level in the circulation system.

Furthermore, hematocrit levels of different animal groups were examined. The hematocrit value in the INas and IVen administrated group were both significantly higher compared with the normal control (*p* < .01, *n* = 10; Figure 1(E)). Of note, the hematocrit value in the INas administrated group was significantly lower than that in the IVen treated group (*p* < .01, *n* = 10). These findings suggested that the INas delivery of EPO would cause less systemic side effects.

### INas delivery of EPO mediated functional rescue

**Figure 2.**
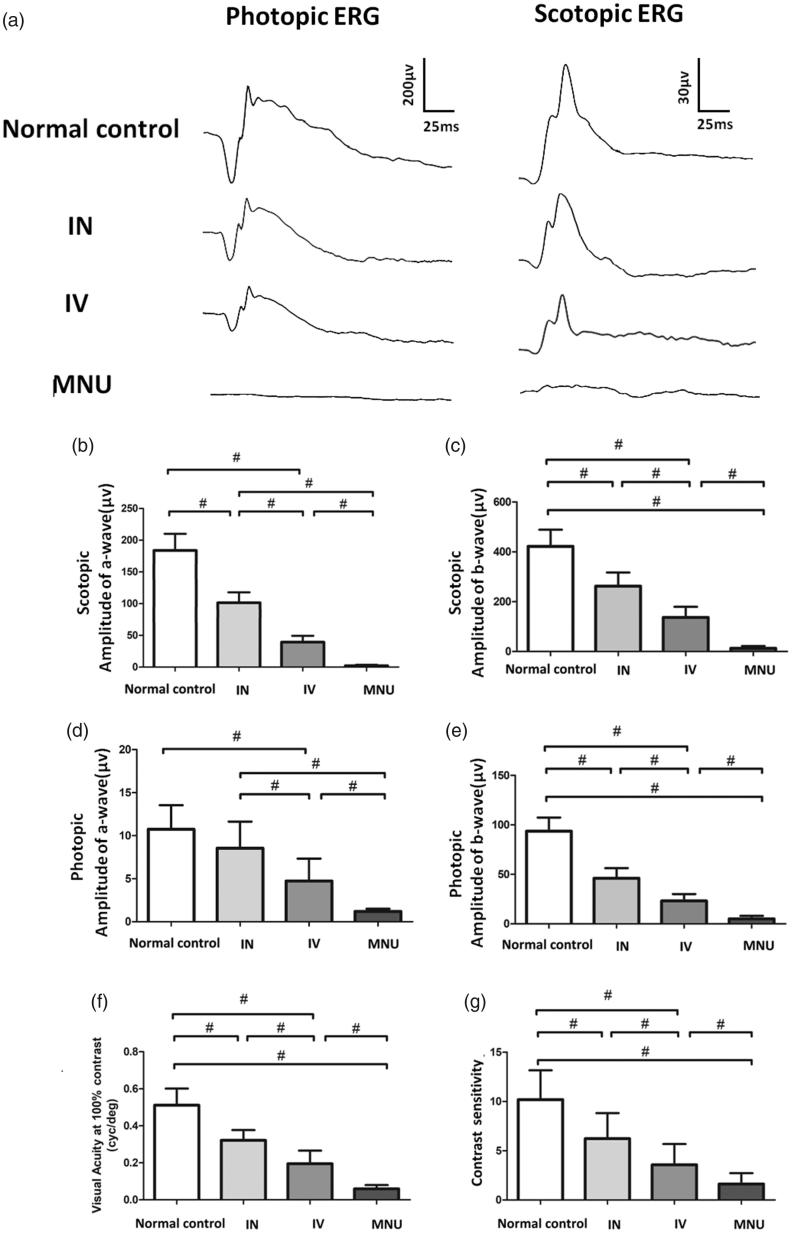
(A) Representative ERG responses of mice from different animal groups. (B) and (C) Scotopic a- and b-wave amplitudes in the MNU group were significantly smaller than that in the normal control group. The scotopic a- and b-wave amplitudes in the INas administered group was significantly larger than those in the IVen administered group. (D) and (E) The photopic a- and b-wave amplitudes in the MNU group were significantly smaller than those in the normal control group. The photopic a-wave amplitudes in the INas administered group was larger than that in the IVen administered group, however, the difference was not significant. The photopic b-wave amplitudes in the INas administered group was significantly larger than that in the IVen administered group, suggesting the INas delivery of EPO conferred pronounced protection on the ERG function of the MNU administered mice. (F) The mice in the IVen administered group responded better to the raster stimulus than those mice in the MNU group. The visual acuity in the IVen administered group was significantly larger than that in the MNU group. The visual acuity in the INas administered group was significantly larger compared with the IVen administered group. (G) The INas group had a contrast sensitivity significantly larger compared with the IVen administered group (ANOVA analysis followed by Bonferroni's post-hoc analysis was performed, #*p* < .01, for differences between groups; *n* = 10).

Experimental animals were subjected to functional examination and their ERG responses to flash stimulus are shown in Figure 2(A). Typical ERG waveforms were evident in the normal controls. Conversely, the ERG responses of the MNU group were terribly abolished. The scotopic a- and b-wave amplitudes in the MNU group were significantly smaller than those in the normal control group (*n* = 10; *p* <.01; Figure 2(B,C)). The scotopic ERG responses in both INas and IVen administered group were effectively preserved. The scotopic a- and b-wave amplitudes in the INas administered group were significantly larger than those in the IVen administered group (*p* < .01; *n* = 10). Furthermore, the photopic a- and b-wave amplitudes in both the INas and IVen administered group were significantly larger than those in the MNU group (*p* < .01; *n* = 10; Figure 2(D,E)). The photopic b-wave amplitude in the INas administered group was ⁓50.3% of the normal controls, while the IVen administered group was ⁓36.1% of the normal controls. ERGs could reveal the function of different components in visual pathways, with a-wave reflects the response of photoreceptors and b-wave reflects the electrophysiological activity of bipolar cells (Neveling et al., 2013). Our data suggested that the INas delivery of EPO conferred pronounced protection on the ERG function of MNU administered mice.

In order to determine whether the electrophysiological protection leads to vision improvements in a behavioral sense, we tested the experimental animals by the vision-guided optokinetic tests. The visual acuity in normal control group was significantly larger than that in the MNU group(*p* < .01; *n* = 10; Figure 2(F)). The EPO administered mice responded better to the raster stimulus than those in the MNU group. The visual acuity in the IVen administered group was significantly larger than that in the MNU group (*p* < .01; *n* = 10). The visual acuity in the INas administered group was significantly larger than that in the IVen administered group. Furthermore, the mice in the INas group had a contrast sensitivity significantly larger than that in the IVen administered group (*p* < .01; *n* = 10; Figure 2(G)). These findings suggested that the mice in the INas administered group had better optokinetic performance compared with the IVen administered group.

### INas delivery of EPO mediated structure rescue

**Figure 3.**
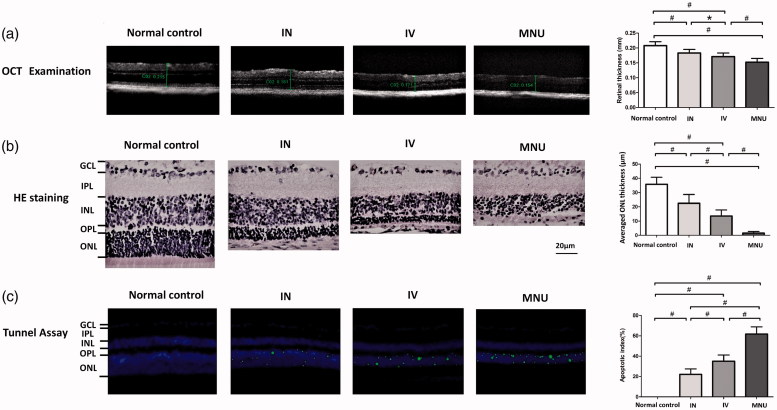
(A) The OCT examination found that the ONL architecture in the MNU group was terribly disrupted. However, these mice in the IVen and INas administered group had relatively intact retinal architecture. The retinal thickness in the MNU group was significantly smaller than that in the IVen administered group. The retinal thickness in the IVen administered group was significantly smaller than that in the INas administered group. (B) The mean ONL thickness in the IVen administered group was significantly smaller than that in the INas administered group. (C) TUNEL-positive cells in the INas administered group was significantly less compared with the MNU group. The apoptotic index (AI) of the MNU group was significantly larger than that of the IVen administered group. The AI of the INas group was significantly smaller compared with the IVen administered group (GCL, Ganglion cell layer; IPL, inner plexiform layer; OPL, outer plexiform layer; ONL, outer nuclear layer; INL, inner nuclear layer; ANOVA analysis followed by Bonferroni's post-hoc analysis was performed,*p < .05, #*p* < .01, for differences between groups; *n* = 10).

The OCT examination was performed to analyze the retinal structure in vivo. OCT examination showed that the ONL architecture in the MNU group was terribly disrupted. On the other hand, the mice in the IVen and INas administered group had relatively intact retinal architecture (Figure 3(A)). Quantification analysis showed that the retinal thickness in the MNU group was significantly smaller than that in the IVen administered group (*p* < .01; *n* = 10). The retinal thickness in the INas administered group was significantly larger than that in the IVen administered group (*p* < .05; *n* = 10).

On closer inspection, the retinal sections were assessed under light microscopy. The retina in the MNU group was clearly thinned relative to the normal control group (Figure 3(B)). The retinas of the INas administered group maintained a substantial proportion of ONL. Quantification analysis showed that the mean ONL thickness of the IVen administered group was significantly larger than in the MNU group (*p* < .01; *n* = 10). The mean ONL thickness in the IVen administered group was significantly smaller compared with INas administered group (*p* < .01; *n* = 10). Furthermore, TUNEL assay was performed to examine the retinal apoptotic status (Figure 3(C)). The TUNEL-positive cell was rarely detected in the retinas of the normal control group. On the other hand, numerous TUNEL -positive cells were found in the ONL of the MNU group, suggesting that MNU toxicity induced massive photoreceptor apoptosis. The TUNEL-positive cells in the INas administered group were remarkably less compared with the MNU group. Subsequently, the apoptotic index (AI) was analyzed to quantify the apoptotic status. The AI of the MNU group was significantly larger than that in the IVen administered group (*p* < .01; *n* = 10). The AI of the INas group was significantly smaller than the IVen administered group (*p* < .01; *n* = 10), suggesting that the INas delivery of EPO ameliorated the photoreceptor apoptosis in MNU administered mice.

### Regional cone cell counts in the retinas of the treated mice

**Figure 4.**
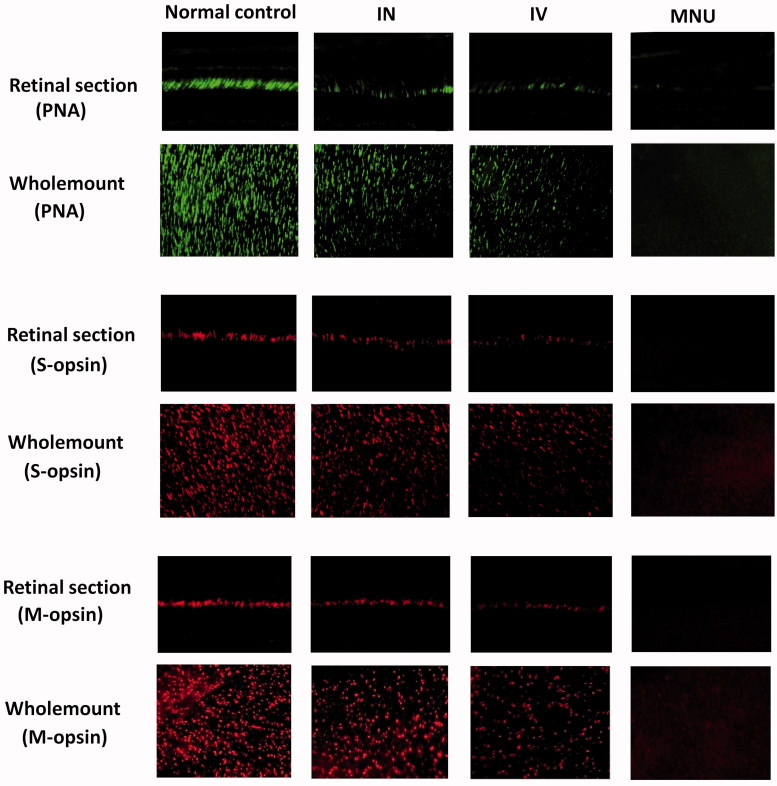
In the normal control mice, PNA staining was evident at the outer segments of photoreceptors. A pronounced proportion of PNA staining was retained in the retinal sections of the INas administered group. Conversely, no PNA staining was found in the retinal sections of the MNU group. The cone photoreceptors in the dorsal-temporal (DT) and dorsal-nasal (DN) quadrants were preferentially preserved by INas delivery of EPO. The M- and S- opsin positive cells were lost in the retinal section and whole mount of the MNU group. On the other hand, the M- and S-opsin positive cells were evident in the retinal section and whole mount of the INas administered group. The M- and S--opsin staining of the IVen group was prominently weaker than that of the INas administered group. In the INas group, the M- and S--opsin positive cells were distributed throughout the retinal whole mount. However, the distribution was not uniformly equal and formed a topographic gradient across retina: the highest number of M-opsin positive cell was in the DT quadrants and the fewest cell in the ventral-nasal VN quadrant; the highest number of S-cone opsin positive cell was in the VN quadrants and the fewest cell in the DT quadrant.

Typically, the cone photoreceptor in mouse retina degenerated rapidly. Most of the cone opsins disappeared within one-week post-MNU administration. In order to decide whether the cone photoreceptors were rescued by EPO therapy, we performed immunostaining works to verify the viability of cone photoreceptors. In the retinal sections of normal controls, PNA staining was evident at the outer segments of photoreceptors (Figure 4). Conversely, no PNA staining was found in the retinal sections of the MNU group. A pronounced proportion of PNA staining was retained in the retinal sections of the INas administered group. Subsequently, the density of PNA staining in the retinal whole-mounts was measured. The average PNA-positive cell counts in the INas administered group was significantly larger than that in the MNU group (*p* < .01; *n* = 10; Table 1), suggesting that the cone photoreceptors were effectively rescued by EPO therapy. Meanwhile, the average PNA-positive cell counts in the INas administered group was significantly larger compared with IVen administered group (*p* < .01; *n* = 10). In whole-mount works of the INas administered group, the PNA-positive cell counts of the ventral-nasal (VN) quadrant was the smallest among the four retinal quadrants. On the other hand, the cone photoreceptors in the dorsal-temporal (DT) and dorsal-nasal (DN) quadrants were preferentially preserved by INas delivery of EPO.

**Table 1.** Cell counts in different quadrants of retinal whole mounts.

Location	PNA-positive cell counts			
	Control Group	IN	IV	MNU
ST	1729 ± 121^‡§∮^	12161 ± 81^†^^§∮^	733 ± 66^†^^‡∮^	33 ± 8^†^^‡§^
IT	1615 ± 116^‡§∮^	862 ± 70^†^^§∮^	440 ± 55^†^^‡∮^	19 ± 6^†^^‡§^
C	1638 ± 109^‡§∮^	410 ± 61^†^^§∮^	206 ± 42^†^^‡∮^	8 ± 3^†^^‡§^
IN	1679 ± 104^‡§∮^	423 ± 58^†^^§∮^	239 ± 36^†^^‡∮^	3 ± l^†^^‡§^
SN	1692 ± 113^‡§∮^	992 ± 73^†^^§∮^	466 ± 49^†^^‡∮^	26 ± 7^†^^‡§^
Average	1670 ± 118^‡§∮^	780 ± 67^†^^§∮^	417 ± 48^†^^‡∮^	18 ± 8^†^^‡§^
	S-opsin positive cell counts			
ST	220 ± 38^‡§∮^	106 ± 28^†^∮	81 ± 19^†^∮	0^†^^‡§^
IT	569 ± 51^‡§∮^	294 ± 29^†^^§∮^	160 ± 27^†^^‡∮^	0^†^^‡§^
C	871 ± 72^‡§∮^	401 ± 55^†^^§∮^	280 ± 39^†^^‡∮^	0^†^^‡§^
IN	956 ± 76^‡§∮^	588 ± 51^†^^§∮^	366 ± 45^†^^‡∮^	9 ± 4^†^^‡§^
SN	870 ± 62^‡§∮^	429 ± 40^†^^§∮^	311 ± 41^†^^‡∮^	6 ± 2^†^^‡§^
Average	697 ± 66^‡§∮^	362 ± 42^†^^§∮^	239 ± 37^†^^‡∮^	3 ± 2^†^^‡§^
	M-opsin positive cell counts			
ST	1233 ± 88^‡§∮^	890 ± 72^†^^§∮^	399 ± 51^†^^‡∮^	13 ± 5^†^^‡§^
IT	1179 ± 82^‡§∮^	529 ± 60^†^^§∮^	313 ± 48^†^^‡∮^	8 ± 3^†^^‡§^
C	1171 ± 85^‡§∮^	300 ± 53^†^^§∮^	195 ± 37^†^^‡∮^	0^†^^‡§^
IN	1199 ± 86^‡§∮^	287 ± 51^†^^§∮^	176 ± 40^†^^‡∮^	0^†^^‡§^
SN	1210 ± 83^‡§∮^	573 ± 62^†^^§∮^	336 ± 57^†^^‡∮^	10 ± 6^†^^‡§^
Average	1198 ± 84^‡§∮^	516 ± 65^†^^§∮^	283 ± 48^†^^‡∮^	6 ± 4^†^^‡§^

† p < .05 for difference compared with control group.

^‡^* *p* < .05 for difference compared with IN administered group.

§ *p* < .05 for difference compared with IV-administered group.

∮ *p* < .05 for difference compared with MNU group.

All values are presented as mean ± SD; *n* = 10 per group.

In greater detail, the viability of different cone populations was examined using opsin-specific antibodies. As shown in the retinal sections, both M- and S-opsin positive cells in the MNU group were lost. On the other hand, the positive M-opsin and S-opsin positive cells were evident in the retinal sections of the INas administered group, although with a delayed manner relative to the normal controls. The M-opsin and S-opsin staining in the retinal sections of the IVen administered group were weaker compared with the INas administered group. The average number of M-cone opsin-positive cells in the INas group and IVen administered group was respectively 43.0% and 23.6% of that in the normal control (Table 1); the average number of S-cone opsin–positive cells in the INas and IVen administered group was 51.9% and of 34.2% respectively, of that in the normal control. In the INas administered group, both the M- and S-cone opsin–positive cells were distributed throughout the retinal whole-mount. However, the distribution was not uniformly equal and formed a topographic gradient across retina: most of the M-opsin positive cells located in the DT quadrants, and the fewest in the VN quadrant; most of the S- opsin positive cells located in the VN quadrants, and the fewest in the DT quadrant. These findings suggested the M-cone, and S-cone populations were both amenable to the INas administration of EPO. In particular, the M-cone photoreceptors in the DT quadrant and S- cone photoreceptors in the VN quadrant benefited most from the EPO therapy.

### Mechanisms responsible for the protection

**Figure 5.**
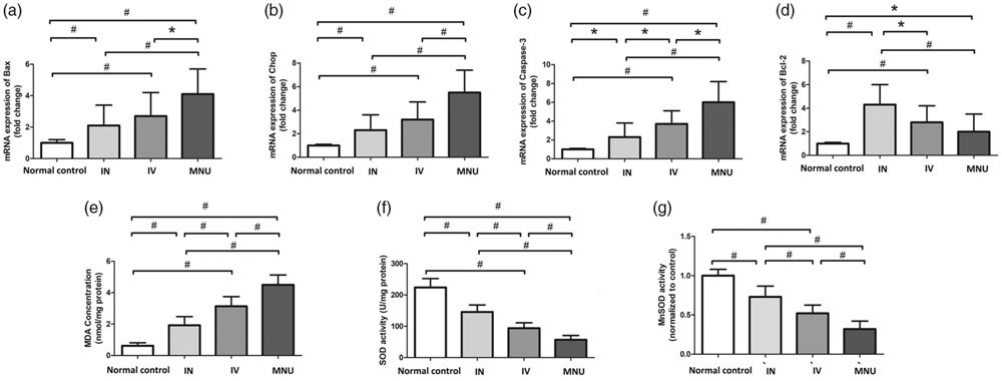
(A–C) The qRT-PCR assay showed that the mRNA levels of Caspase-3, CHOP, and Bax in the INas administered group were significantly down-regulated compared with the MNU group. The mRNA level of Caspase-3, CHOP, and Bax in the INas administered group was also significantly lower than that in the IVen administered group. (D) In the INas administered group, the mRNA level of Bcl-2 was significantly higher compared with the MNU group, suggesting that the anti-apoptotic mechanism should be responsible for the EPO mediated protection. (E) The retinal MDA concentration in the INas administered group was significantly lower than that in the MNU group. (F) The expression level of SOD in the INas administered group was significantly up-regulated compared with the MNU group, suggesting that the INas delivery of EPO could alter the oxidation status in the degenerative retina. (G) The Mn-SOD level in the IVen administered group was significantly higher than that in the MNU group. Meanwhile, the Mn-SOD level in the IVen administered group was significantly lower than that in the INas administered group (ANOVA analysis followed by Bonferroni's post-hoc analysis was performed,*p < .05, #*p* < .01, for differences between groups; *n* = 10).

The qRT-PCR was performed to analyze the mechanisms underlying the EPO induced protection. The mRNA levels of Caspase-3, CHOP, and Bax in the INas administered group was significantly down-regulated compared with the MNU group, (*p* < .01; *n* = 10; Figure 5 (A–C)). The mRNA level of Caspase-3, CHOP, and Bax in the INas administered group was also significantly lower than that in the IVen administered group (*p* < .05; *n* = 10). On the other hand, the mRNA level of Bcl-2 in the INas administered group, was significantly higher compared with the MNU group (*p*< .05; *n* = 10; Figure 5(D)). These findings suggested that the anti-apoptotic mechanism was, at least partly, responsible for the EPO mediated protection. MDA is a reactive electrophile species that caused by the peroxidation of polyunsaturated fatty acids. It acts as a presumptive marker of lipid peroxidation during photoreceptor degeneration (Farmer & Davoine, 2007). The retinal MDA concentration in the INas administered group was significantly lower compared with the MNU group (*p* < .01; *n* = 10, Figure 5(E)).On the other hand, the expression level of SOD, an endogenous antioxidant in the INas administered group was significantly higher compared with the MNU group (*p* < .01; *n* = 10; Figure 5(F)). Manganese superoxide dismutase (MnSOD) is a mitochondrial protein known as a potent antioxidant defense against superoxide radicals that produced by the electron transport chain (Ansenberger-Fricano et al., 2013). The Mn-SOD level in the IVen administered group was significantly higher than that in the MNU group (*p* < .01; *n* = 10; Figure 5(G)), suggesting the EPO conferred profound mitochondrial protection during MNU induced photoreceptor apoptosis. Meanwhile, the Mn-SOD level in the IVen administered group was significantly lower than that in the INas administered group (*p* < .01; *n* = 10). Collectively, these findings suggested that the INas delivery of EPO could alter the oxidation status in the degenerative retina.

## Discussion

Inherited retinal dystrophies generally lead to impaired visual function and photoreceptor death (Cronin et al., 2007). Given the escalating prevalence of these retinopathies, efficient drug delivery to the posterior segments of eye is desirable. In clinical practice, invasive methods such as intraocular injection always cause structural impairments to the eyeball and result in complications that are even more terrible than the retinopathy itself (Thrimawithana et al., 2011). On the other hand, it is difficult to reach effective drug concentrations in retinas via topical administration due to the consolidated anatomic barriers (Fangueiro et al., 2016). To date, there is no safe and patient-friendly drug delivery approach that is suitable for retinal diseases. Recently, increasing interests are placed in the possibility of delivering therapeutic agents through the INas route (Garcia-Rodriguez & Sosa-Teste, 2009; De Rosa et al., 2005). In this context, we harnessed the INas delivery to build up the EPO therapeutic strategy.

Our results showed that the INas delivery is efficient to enhance the bioavailability of EPO in retinas, and afforded pronounced protective effects on the degenerative retinas. To our knowledge, this is the first study the protective effects of INas delivered EPO on the retinopathy. Admittedly, the etiology of the MNU induced photoreceptor degeneration is somewhat different from that occurs in RP patients. The MNU induced photoreceptor degeneration is rapidly progressing, while the RP is a relatively chronic retinopathy in human patients. Although the outcome of the MNU administration partially resembles RP, the mechanistic underpinning and kinetics are very different. Moreover, the following improvements are integrated into the methodology of our work. First, these *in vivo* examination approaches are used to test the efficacy of treatment. For example, SD-OCT examination can measure the retinal thicknesses noninvasively, affording a valuable tool to score the therapeutic efficacy without killing the animals. These advantages would reduce dramatically the number of experimental animals that are required in the screening tests. Moreover, traditional therapeutic trials only describe the viability of overall photoreceptors, potentially obscuring accurate information for a single photoreceptor population. Herein, we dissect the protective effects of EPO administration on the rod and cone photoreceptors respectively. The immunostaining experiments suggest that both the M-cone and S-cone populations in the degenerative retinas are rescued by INas delivered EPO. Particularly, the immunostaining studies based on retinal whole-mounts allow us to quantify the regional cell viability comprehensively. Although the retinal structure of mouse eye is somewhat different from that in human, our findings lay the groundwork for future development of EPO pharmaceuticals that suitable for INas administration.

EPO a versatile molecule with neuroprotective effects (Maiese, 2016; Busch et al., 2014). Given the retina is an extended component of the central nervous system, it comes as no surprise that EPO might play a significant role in the retinal homeostasis. Several pioneering experiments have demonstrated that the intraocular delivery of EPO can protect against retinal degeneration (Rex et al., 2004; Colella et al., 2011). The present study shows that INas delivery is more efficient and the induced beneficial effects are more robust in comparison with the IVen delivery. Several mechanisms should be responsible for the potency of INas delivery. Generally, a quantity of the EPO can be absorbed across the nasal mucosa and reach the systemic circulation from where it will cross the blood-retina barrier. However, the efficiency of this pathway is highly limited and dependent on the molecular weight of the drug (Merelli et al., 2011). Apart from the rapid nasal absorption, olfactory region has unique anatomic and pathological attributes which would define the extracellular and intracellular routes to orbital cavity (Capsoni et al., 2009; Takahashi et al., 2010). Olfactory epithelium is located just below the cribriform plate that separates the nasal cavity from orbital cavity. Several vascular and nerves penetrate into the orbital cavity through small holes in the cribriform plate (Illum, 2002; Robert et al., 2016; White et al., 2005). For example, the anterior and posterior ethmoidal branches of the ophthalmic artery cross this region and supply blood for the olfactory epithelium. Moreover, the bundle of nerve terminals that constitute the olfactory tract also passes through the holes of the cribriform plate. Depending on a direct anatomic connection between the superior turbinate and the orbital cavity, a substantial amount of INas delivered EPO would readily transverse the cribriform plate and reach the orbital cavity (Garcia-Rodriguez & Sosa-Teste, 2009; Frey et al., 1997; Thorne et al., 1995). Additionally, an insightful study shows that INas delivered drugs can be transported to the eyeball via trigeminal-associated pathway (Thorne et al., 2004). As the olfactory pseudo epithelium is innervated by the trigeminal nerve, the INas administered drug can reach the trigeminal nerve and perineural space after absorption (Johnson et al., 2010).

Concentrations of the INas administered drug in the trigeminal nerve and optic nerve are significantly higher than the other connected structures such as the olfactory bulbs, olfactory tubercle, striatum etc., (Yang et al., 2009). These findings suggest that the trigeminally innervated structures such as eye also receive drug from the trigeminal nerve (Guo et al., 2016). Consequently, a combination of these pathways would collectively contribute to the INas delivery of drugs to eye.

Systemic EPO administration always increases the hematocrit value of the subjects and further elevates the risk of thrombosis (Wiessner et al., 2001). However, INas delivered EPO is advantageous since it bypasses systemic/hepatic extraction (Liu et al., 2001). As shown in the present study, the INas delivery of EPO would exert much less unwanted side effects on the subjects. The hematocrit level in the INas administered group is significantly lower than that in the IVen administered group. Meanwhile, the retinal EPO level in the INas administered group is significantly higher than that in the IVen administered group. Another study also shows that the peripheral organs of the systemic administered rats had drug concentrations up to four times greater than those peripheral organs of the INas administered rats (Yang et al., 2009). Accordingly, the INas delivery could avoid the unwanted side effects and increase the bioavailability of EPO.

In conclusion, our study shows the INas delivery of EPO could alleviate photoreceptor apoptosis and visual impairments in the MNU administered mice. Compared with IVen delivery, the INas delivery is more potent and efficient. Together with its noninvasive nature, the INas delivery is readily acceptable for out-patient use. These findings highlight the possibility that the INas delivery of EPO might be developed into a viable therapeutic option for retinopathies require repeated intervention.
